# IKBKE, a prognostic factor preferentially expressed in mesenchymal glioblastoma, modulates tumoral immunosuppression through the STAT3/PD‐L1 pathway

**DOI:** 10.1002/ctm2.130

**Published:** 2020-07-23

**Authors:** Li Yi, Gaochao Guo, Jiabo Li, Xiaoguang Fan, Tao Li, Luqing Tong, Peidong Liu, Xuya Wang, Feng Yuan, Shengping Yu, Qiang Huang, Xuejun Yang

**Affiliations:** ^1^ Department of Neurosurgery Tianjin Medical University General Hospital Tianjin China; ^2^ Laboratory of Neuro‐Oncology Tianjin Neurological Institute Tianjin China; ^3^ Department of Oncology‐Pathology, Karolinska Institutet Karolinska University Hospital Solna Stockholm Sweden; ^4^ Department of Neurosurgery, Henan Provincial People's Hospital People's Hospital of Zhengzhou University Zhengzhou Henan China; ^5^ Department of Neurosurgery Johns Hopkins University School of Medicine Baltimore MD USA

**Keywords:** glioma, IKBKE, immunosuppression, mesenchymal subtype

## Abstract

Inhibitor of nuclear factor kappa‐B kinase subunit epsilon (IKBKE) plays critical roles in the proliferation, invasion, and epithelial‐mesenchymal transition (EMT) of glioblastoma (GBM). However, as an immune response factor, few studies have focused on the role of IKBKE in the glioblastoma‐mediated immunosuppressive microenvironment. Here, we found a higher IKBKE expression level in gliomas corresponding to higher malignancy of the tumor. The highest level of IKBKE expression was examined in the core region of GBM tissues as well as the mesenchymal subtype, which are featured with necrosis, immunocyte infiltration, and immunosuppression. Further in silico analysis demonstrated that the JAK/STAT as the signaling pathway most associated with IKBKE in mesenchymal GBM. The co‐expression patterns of IKBKE, pSTAT3, and PD‐L1 were detected within GBM tissues. Mechanistically, IKBKE could interact with STAT3 and thus enhancing the phosphorylation level of STAT3 and its nuclear translocation. In addition, pSTAT3 could transcriptionally regulate the expression of PD‐L1 through binding to its promoter. In vivo results further confirmed the inhibitory effect of the IKBKE downregulation on tumor growth. Collectively, our findings suggest IKBKE as the central node in the crosstalk between NF‐κB and STAT3 signaling within mesenchymal GBM. Targeting GBM through inhibiting IKBKE could restrain tumor growth and tumor‐mediated immunosuppressive environment.

## Graphical abstract

1. Higher IKBKE expression was detected in higher grades of glioma.

2. IKBKE is enriched in mesenchymal GBM, which confers immunosuppressive signature.

3. IKBKE correlates with JAK/STAT signaling in MES GBM.

4. IKBKE activates the phosphorylation of STAT3, which transcriptionally regulates PD‐L1 expression.

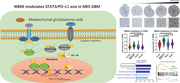

**Abbreviations**
AUC: area under the curve
CGGA: Chinese Glioma Genome Atlas
CL: classical
co‐IP: co‐immunoprecipitation
DEGs: differential expressed genes
EM: enhanced margin
EMT: epithelial‐mesenchymal transition
ER: enhancing region
GBM: glioblastoma multiforme
IHC: Immunohistochemistry
IKBKE: inhibitor of nuclear factor kappa‐B kinase subunit epsilon
IKK: IκB kinase
Ivy GAP: Ivy Glioblastoma Atlas Project
KEGG: Kyoto Encyclopedia of Genes and Genomes
MES: mesenchymal
MSigDB: Molecular Signatures Database
NE: neural
NR: necrotic region
PN: proneural
STAT3: signal transducer and activator of transcription 3
TCGA: The Cancer Genome Atlas
TMA: tissue microarray
TNBCs: triple‐negative breast cancers

## 1 INTRODUCTION

Glioblastoma also known as glioblastoma multiforme (GBM) represent the most frequent form of primary intracranial malignancies, and are characterized by extensive heterogeneity at histological and molecular levels. Despite recent advances in cancer management, the overall outcomes for GBM patients remains modest with only 14.6 months median survival time^1^ and 5.6% 5‐year survival rate.^2^ Therefore, an understanding of its molecular mechanisms and the development of effective targeted therapies for GBM is still needed.

Inhibitor of nuclear factor kappa‐B kinase subunit epsilon (IKBKE), also named IκB kinase epsilon (IKKε), is a member of the IκB kinases (IKKs) family, which responds to stimuli such as TNF‐α, IL‐1, and IL‐17.^3^, ^4^ As the upstream kinase of IκBα, IKKs directly phosphorylates IκBα, triggering its ubiquitylation and degradation, thus release the p65/p50 (NF‐κB) heterodimers that translocate into the nucleus where it can bind to DNA and induce the transcription of target genes, thereby generating corresponding biological functions.^5^ IKBKE has been identified as an oncogene in many human cancers, including pancreatic cancer,^6^ triple‐negative breast cancer,^7^, ^8^, ^9^ and non‐small cell lung cancer.^10^ In gliomas, IKBKE is also involved in tumorigenicity through multiple tumor‐associated pathways and biological processes. It was indicated that the enrichment of IKBKE in glioma contributes to its resistance to apoptosis through the activation NF‐κB pathway.^11^ Our previous research demonstrated that IKBKE could also regulate proliferation, invasion as well as the epithelial‐mesenchymal transition (EMT) in glioma cells through Hippo pathway.^12^, ^13^ However, as an immune response factor, few studies have focused on the role of IKBKE in the glioblastoma‐mediated immunosuppressive microenvironment.

Emerging evidence suggests the signal transducer and activator of transcription 3 (STAT3) as one of the main transcriptional regulators paving the way for cancer growth through inhibition of antitumoral immunity.^14^, ^15^, ^16^, ^17^ Activation via upstream signals, STAT3 undergoes phosphorylation, homo‐dimerization, and translocates to the cell nucleus where they act as an activator,^18^ transcriptionally mediates a variety of downstream target genes that are crucial for tumoral cell growth, invasion, and immune‐escape.^19^, ^20^, ^21^, ^22^, ^23^ The synergic effect of STAT3 and NF‐κB has been reported in cancers previously^24^, ^25^, ^26^ as well as in the phenotype transition of GBM recently.^27^, ^28^ Both JAK/STAT3 and NF‐κB signaling have been strongly implicated in the pathogenesis of mesenchymal GBM^29^, ^30^ and closely related proneural‐mesenchymal phenotype transition.^31^, ^32^ It is considered that the transformation of mesenchymal subtype is mediated by NF‐kB‐dependent master transcription factors like STAT3, C/EBPb, and TAZ, accompanied by the increased percentage of CD44 subpopulations and radioresistant properties.^32^ However, there are remaining knowledge gaps underlying the crosstalk between NF‐κB and STAT3 signaling in GBM‐associated immune suppression.

In this study, we reported that IKBKE is hyperactive in GBM and contributes to the worse prognosis of glioma patients. Mesenchymal GBM, which is characterized by overall necrosis and associated inflammatory infiltrates, owns a higher IKBKE expression and tumor immunosuppressive features. IKBKE could promote tumor‐mediated immunosuppression through phosphorylation of STAT3 and then transcriptionally regulate PD‐L1(CD274) expression. Silencing IKBKE could decrease tumor growth and PD‐L1 expression in vivo and getting overall survival benefits in tumor‐bearing mice. Collectively, our findings confirmed that IKBKE as a prognostic factor that preferentially expressed in mesenchymal glioblastoma and induces tumoral immunosuppression through the STAT3/PD‐L1 pathway.

## 2 MATERIALS AND METHODS

### 2.1 Cell lines and cell culture

Human GBM cell lines U87 and U251 were obtained from Shanghai Institute of Biochemistry and Cell Biology. Dulbecco's modified Eagle's medium (DMEM) supplement with 10% fetal bovine serum (FBS; Gibco, USA) was used for cell culture. The cells were cultured in an incubator containing 5% CO_2_ at 37°C.

### 2.2 Lentiviral transfection

IKBKE shRNA was selected based on previously published articles.^13^, ^33^ We chose the most effective shRNA sequence (5ʹ‐GCATCATCGAACGGCTAAATA‐3ʹ) constructed with lentiviral vector. Scrambled sequence (5ʹ‐TTCTCCGAACGTGTCACGTTTC‐3ʹ) was designed as the negative control (GeneChem, China). The IKBKE plasmid was purchased from Addgene (#15292, Cambridge, USA). The recombinant lentivirus that overexpresses IKBKE was constructed by Shanghai GeneChem (China). The lentiviral transfection was manipulated followed by manufacturer's protocol.

### 2.3 Clinical sample collection

Clinical tissue array and radiological data were obtained from the Department of Neurosurgery at Tianjin Medical University General Hospital (TMUGH). All the samples were histologically graded in accordance with the 2016 World Health Organization (WHO) classification for brain tumors by pathologists. The Ethical Committee of TMUGH granted approval for experiments on human glioma tissues. At TMUGH, the surgeon selected tumors with the criterion as previously described.^34^ For each tumor mass, the tissues were divided into intratumor (necrotic region), tumor border (enhancing margin), and peri‐tumor (enhancing region).

### 2.4 Tissue microarray and immunohistochemistry

The tissue microarray (TMA) collected 55 glioma cases, in which 2 cases were of non‐tumor (cortical dysplasia), 2 cases of Grade I glioma, 12 cases of Grade II glioma, 12 Grade III, and 27 cases of GBM (Grade IV) (Figure S1 and Table S1). The procedure of immunohistochemistry (IHC) was performed as previously described.^35^ Primary antibodies: Anti‐IKBKE antibody (Cell Signaling, USA), Anti‐pSTAT3 (Tyr705) antibody (Cell Signaling, USA) and Anti‐CD274 antibody (Abcam, USA) were incubated with a dilution of 1:100. Secondary antibodies: HRP‐labeled goat anti‐mouse IgG and goat anti‐rabbit IgG were obtained from ZSGB‐Bio, China. The IHC images were observed and acquired though a light microscope (OLYMPUS, Japan).

### 2.5 Immunofluorescence analysis

The immunofluorescent staining was performed as previously described.^35^ GBM frozen tissues were sectioned and fixed with 4% paraformaldehyde fix solution (Beyotime, China). Primary antibodies (pSTAT3 Tyr705 antibody, IKBKE antibody, PD‐L1 antibody) were used with a dilution of 1:100. Alexa‐Fluor 488 labeled donkey anti‐rabbit IgG (Invitrogen, USA, 1:1000) and Alexa‐Fluor 594 labeled donkey anti‐mouse IgG (Invitrogen, USA, 1:1000) were applied to the double‐colored fluorescent staining. Nucleus was labeled by DAPI staining solution (Solarbio, China).

### 2.6 Western blotting analysis

Western blotting was performed rely on relevant protocol. Proteins were transferred onto PVDF membranes (ThermoFisher, USA), and incubated 8 h in the 4°C fridge with the following primary antibodies: Anti‐IKBKE (Cell Signaling, USA, 1:1000), Anti‐CD274 (Abcam, USA, 1:1000), Anti‐pSTAT3 (Tyr705) (Cell Signaling, USA, 1:1000), Anti‐STAT3 (ABclonal, UK, 1:1000), Anti‐GAPDH (ZSGB‐Bio, China, 1:2000). Secondary antibodies: HRP labeled goat anti‐rabbit/mouse IgG (ZSGB‐Bio, China, 1:1000). Chemiluminescent HRP substrate (Millipore, USA) and GBOX system (Syngene Company, UK) were used to detect protein expression.

### 2.7 Plasmids construction and luciferase assay

The promotor sequence of PD‐L1 (CD274) was searched from the NCBI (https://www.ncbi.nlm.nih.gov/) and the corresponding plasmids (STAT3PcDNA/HisC, PcDNA/HisC, CD274pGL3‐Basic, pGL3‐Basic and pRL‐TK) were constructed by Hanbio company. (Hanbio, China) The luciferase reporter assay was performed on U87 and U251 cells through the Dual‐Luciferase Reporter Assay System (E1910, Promega, USA) following the instruction.

### 2.8 Co‐immunoprecipitation

Co‐immunoprecipitation (Co‐IP) analysis was manipulated followed by manufacturer's protocol (#26149, Pierce™ Co‐Immunoprecipitation Kit, Thermo Fisher, USA). Cell lysates were treated with IKBKE rabbit antibody and STAT3 rabbit antibody (Cell Signaling, USA), and a normal rabbit IgG as negative control. Bound proteins were denatured and analyzed by immunoblotting.

### 2.9 Chromatin immunoprecipitation

The chromatin immunoprecipitation (ChIP) assay was manipulated using SimpleChIP Plus Sonication Chromatin IP Kit following manufacturer's protocol (#56383, Cell Signaling, USA). Crosslinked chromatin was immunoprecipitated with anti pSTAT3 rabbit antibody or control rabbit IgG antibody (Cell Signaling, USA). qPCR was performed to quantitative analysis of precipitated DNA. The primers of CD274 (PD‐L1) promoter were as follows: forward primer, 5′‐CAAGGTGCGTTCAGATGTTG‐3′ and reverse primer, 5′‐ GGCGTTGGACTTTCCTGA‐3′.

### 2.10 Animal study

Experiments on mouse were approved by the Ethical Committee of Tianjin Medical University General Hospital. Intracranial xenograft mouse model was established as previously described.^36^, ^37^ U87 cells stably transfected with shControl or shIKBKE lentivirus were injected into mice respectively. Bioluminescence imaging was performed to detect tumoral growth at seventh day after implantation through IVIS Spectrum Live Imaging System (Perkin Elmer, USA).

### 2.11 Bioinformatic analysis

The transcriptomic profile and clinical data were obtained from The Cancer Genome Atlas (TCGA) dataset (https://cancergenome.nih.gov), Chinese Glioma Genome Atlas (CGGA) dataset (http://www.cgga.org.cn) and GSE16011 dataset (www.ncbi.nlm.nih.gov). The pathway enrichment analyses based on Kyoto Encyclopedia of Genes and Genomes (KEGG) were performed using online DAVID database (https://david.ncifcrf.gov). Gene Set Enrichment Analysis (GSEA) was performed according to the software instruction (https://www.gsea-msigdb.org/gsea/index.jsp). The mRNA expression profile of five structures (Leading Edge, Infiltrating Tumor, Cellular Tumor, Microvascular Proliferation, and Pseudopalisading Cells Around Necrosis) within GBM tissues were available from Ivy Glioblastoma Atlas Project (http://glioblastoma.alleninstitute.org/).

### 2.12 Statistical analysis

GraphPad Prism 8.0 (GraphPad Software, USA) was applied for all quantification and statistical analyses. Log‐rank test was applied to compare the survival distributions of two groups in Kaplan‐Meier curves. For bar graphs, unpaired Student's *t*‐test was applied to determine the significance between two groups; one‐way ANOVA was applied for comparisons among three or more groups. *P*‐value of <.05 was regarded as statistically significant.

## 3 RESULTS

### 3.1 IKBKE is elevated in GBM and associated with shorter patient survival

**FIGURE 1:**
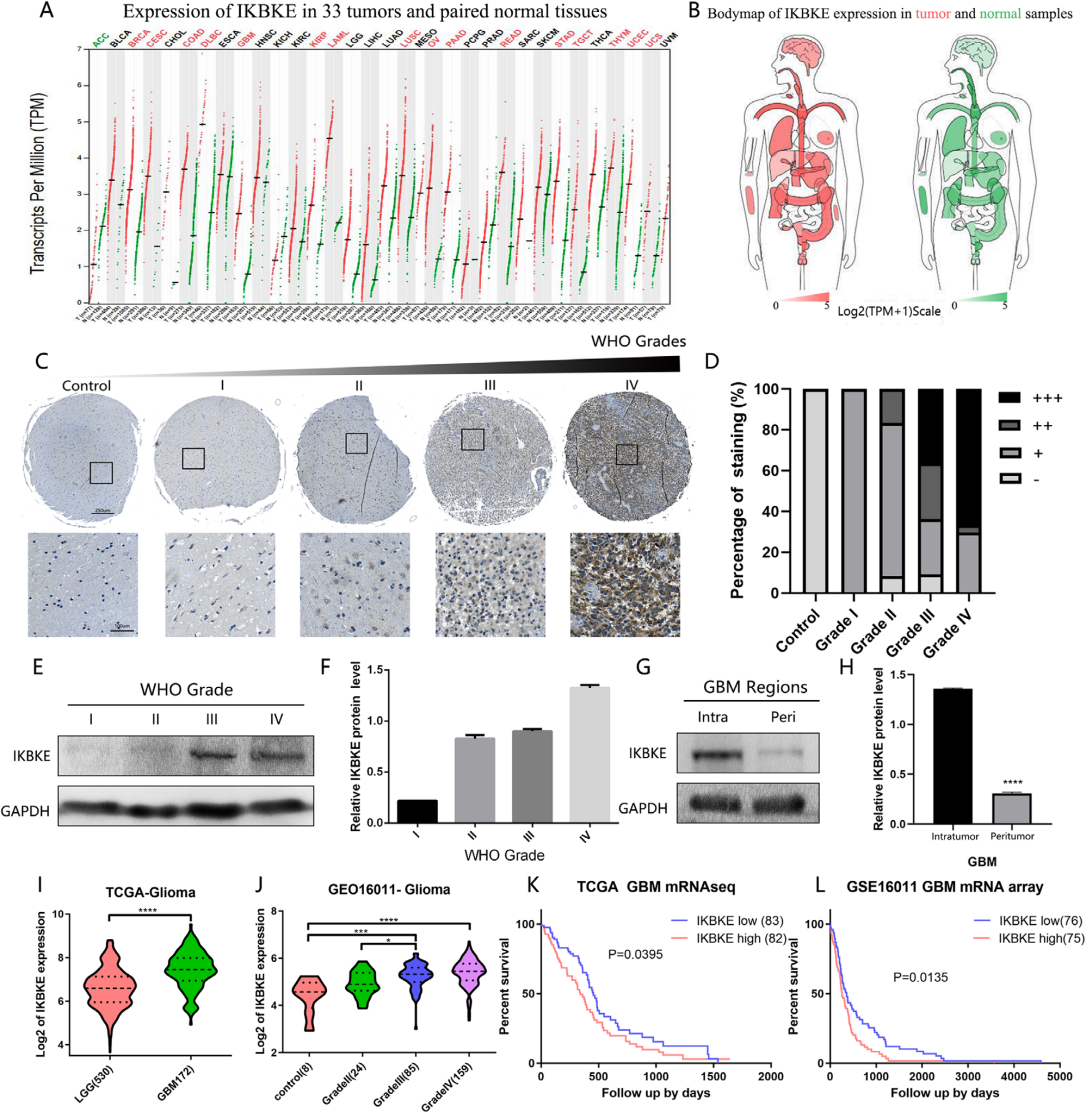
The expression profile of IKBKE in gliomas. A,B, The mRNA expression of IKBKE in 33 tumors and paired control tissues are shown in the dot plot graph and bodymap. C, The Immunohistochemical tissue array stained with IKBKE in gliomas and control brain tissues. (Scale bar = 100 μm). D, Box plot comparing immunostaining scores of IKBKE between tumor and control tissues in the tissue array. E,F, The Western blotting and representative blot of IKBKE in different grades of glioma tissues were shown. G,H, The Western blotting and representative blot of IKBKE expression in the intratumor and peritumor of GBM tissues were shown. I,J, The mRNA expression of IKBKE from the TCGA and GSE16011 datasets in different grades of gliomas. K,L, The Kaplan‐Meier analysis of IKBKE expression with the mean value as the threshold for low or high levels, from the TCGA and GSE16011 datasets in glioblastoma patients. (ns, not significant, *P < .05, **P < .01, ***P < .001, ****P < .0001)

To detect the expression of IKBKE in glioma patients, we extracted mRNA expression data from GEPIA (http://gepia.cancer-pku.cn) (Figure 1A,B; Table S2) to analyze the expression of IKBKE across 33 tumors and paired normal tissues. Compared to other tumors, GBM is one of the tumors that has overexpression of IKBKE. In addition, tissue microarray (TMA) data (Normal control, n = 2; WHO grade I, n = 2; WHO grade II, n = 12; WHO grade III, n = 12; WHO grade IV, n = 27) revealed that IKBKE is increasingly expressed in glioma tissues as the tumor grades increase, and the highest level of IKBKE expression was detected in glioblastomas (Figure 1C,D). Subsequently, western blotting of glioma tissues also showed that the protein expression of IKBKE is enriched in high grade gliomas (WHO Grade III‐IV) (Figure 1E,F), especially in the intratumor of GBM tissues (Figure 1G,H). Gene expression and clinical data from TCGA and GSE16011 cohorts also confirmed these findings, with the significantly higher expression of IKBKE in GBM compared to lower grade gliomas (Figure 1I,J). With regard to Kaplan‐Meier survival curves, the overall survival time in IKBKE high expression GBM patients is significantly shorter than that in IKBKE low expression patients (Figure 1K,L). These data suggest that IKBKE is abnormally activated in GBM and confers poor survival of glioma patients.

### 3.2 IKBKE is enriched in necrotic region of GBM and related to tumor recurrence

**FIGURE 2:**
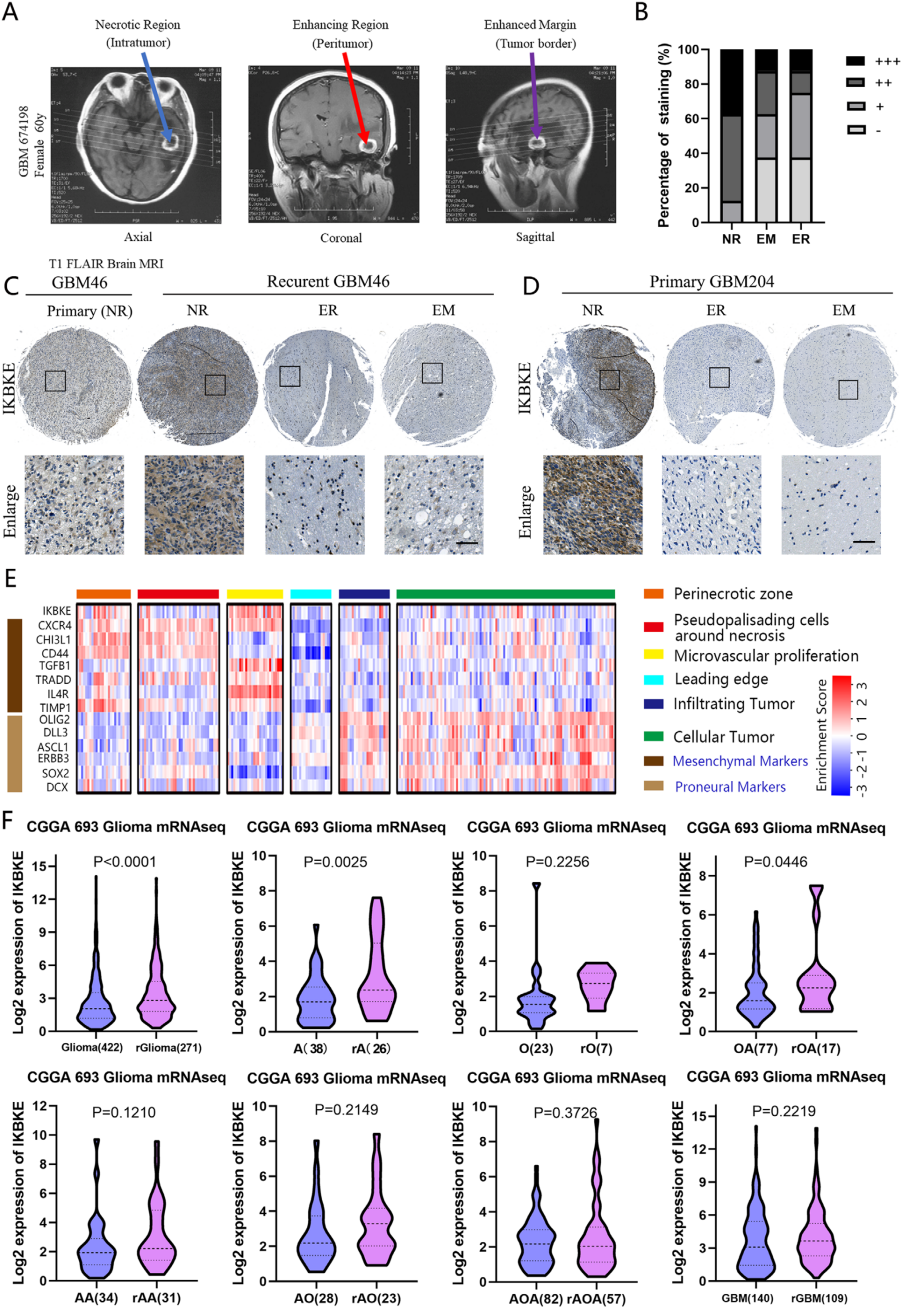
IKBKE is enriched in necrotic region of GBM and related to tumor recurrence. A, T1 FLAIR MRI of different reconstruction planes showing multiple regions within glioblastoma. B, Immunostaining score of IKBKE in three multiregional glioblastoma samples. C,D, The immunohistochemical staining of IKBKE in the multiple regions of primary and recurrent glioblastoma tissue array (Scale bar = 100 μm). E, Heat map showing *z*‐scores of each glioblastoma subtype signature, normalized within each patient sample in the Anatomic Structure Study dataset (Screen of five structures of 122 mRNA samples within 10 patients) from the Ivy GAP database. F, The mRNA expression between primary and recurrent gliomas in different histological subtypes from CGGA dataset. Abbreviations: rGlioma, recurrent glioma; A, astrocytoma; rA, recurrent astrocytoma; O, Oligodendroglioma; rO, recurrent oligodendroglioma; OA, oligoastrocytoma; rOA, recurrent oligoastrocytoma; AA, anaplastic astrocytoma; rAA, recurrent anaplastic astrocytoma; AO, anaplastic oligodendroglioma; rAO, recurrent anaplastic oligodendroglioma; AOA, anaplastic oligoastrocytoma; rAOA, anaplastic oligoastrocytoma; GBM, glioblastoma; rGBM, recurrent glioblastoma

Intratumoral heterogeneity contributes to disease progression,^11^, ^38^ so for a better understanding of the expression pattern of IKBKE in the bulk of tumor, we identified specific tumor microenvironments including the enhancing region (ER), the necrotic region (NR), and the enhanced margin (EM) by image‐guided multiregional glioblastoma sampling (Figure 2A). Through immunohistochemical staining, we observed that IKBKE is significantly increased in the necrotic region of a tumor mass compared to the enhancing region and the enhanced margin (Figure 2B‐D). Moreover, in a case of recurrent GBM, it was demonstrated that the expression level of IKBKE was been upregulated upon recurrence (Figure 2C). To validate our observations in a larger cancer cohort, we retrieved the regional microdissection RNA‐seq data of 37 glioblastomas from Ivy Glioblastoma Atlas Project (Ivy GAP) database (http://glioblastoma.alleninstitute.org/). Confirming our findings from the immunohistochemistry results of multiregional tumor tissues, IKBKE and mesenchymal markers (CXCR4, CHI3L1, CD44, TGFB1, TRADD, IL4R, and TIMP1) are more likely expressed in perinecrotic and microvascular proliferative zones, while the cellular tumor, leading edge and infiltrating tumor regions expressed proneural markers (OLIG2, DLL3, ASCL1, ERBB3, SOX2, and DCX; Figure 2E). The mRNA expression value of 693 gliomas in CGGA dataset also confirmed the previous finding, showing that the expression of IKBKE in recurrent gliomas are significantly higher than that in primary gliomas. If we divide these gliomas by histological subtype, a significant increase in IKBKE expression is also detected in recurrent astrocytoma and oligoastrocytoma, compared to their primary forms (Figure 2F).

### 3.3 IKBKE is preferentially expressed in mesenchymal GBM

**FIGURE 3:**
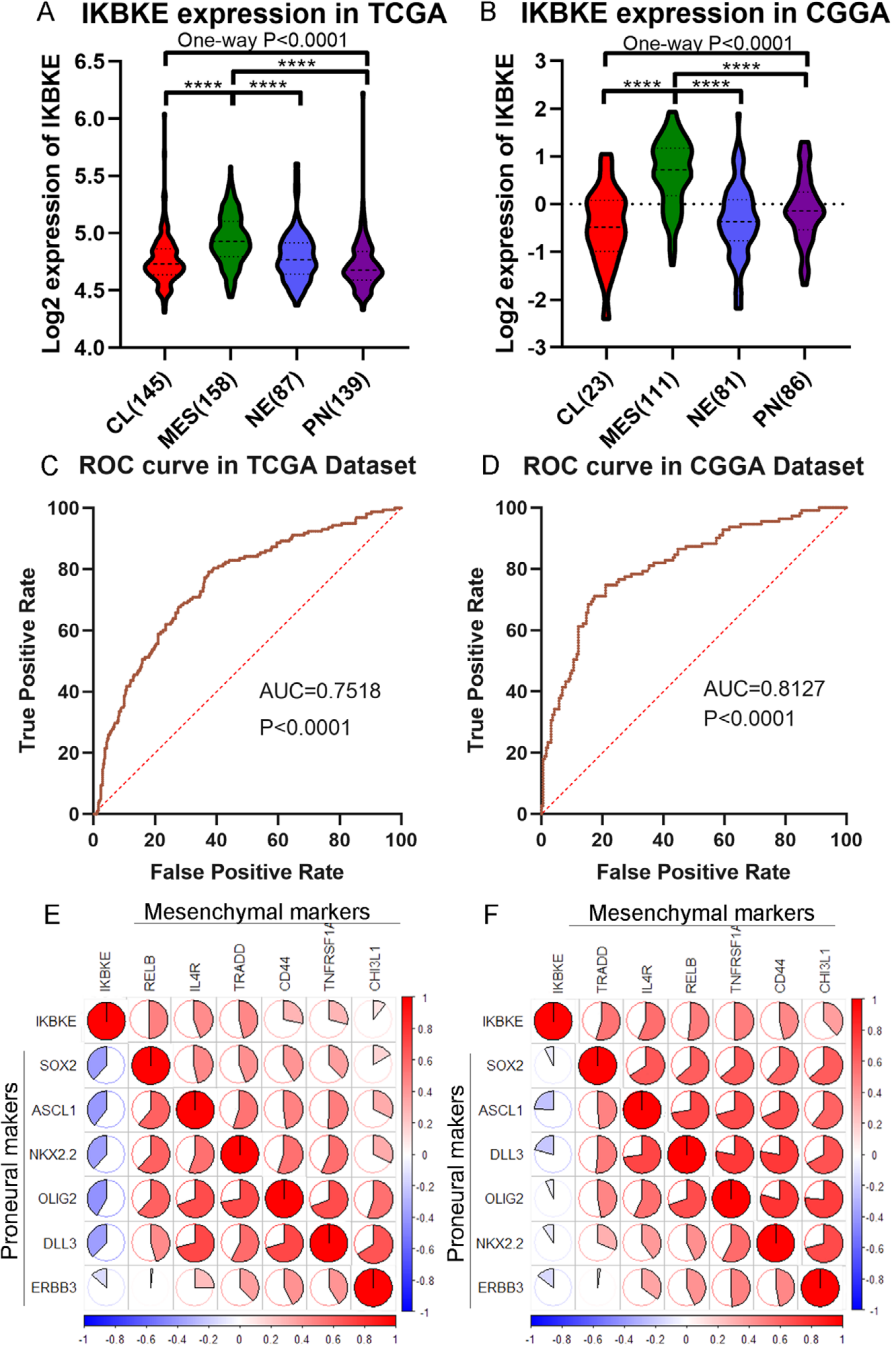
IKBKE is preferentially expressed in mesenchymal GBM. A,B, The mRNA expression of IKBKE in four subtypes of GBM from TCGA and CGGA datasets (****P < .0001). C,D, ROC curve analysis showed the sensitivity and specificity of IKBKE to predict mesenchymal subtype in TCGA and CGGA database. E,F, The relationship between IKBKE and mesenchymal or proneural markers in TCGA and CGGA datasets. The correlation between IKBKE and subtype markers was performed by Pearson correlation analysis

To further explore the molecular relevance between IKBKE and glioma, we analyzed IKBKE expression in different molecular subtypes (classical [CL], mesenchymal [MES], neural [NE], and proneural [PN]) defined by the TCGA network. The data from TCGA and CGGA datasets showed that IKBKE is significantly enriched in mesenchymal subtype among four subtypes of GBM (Figure 3A,B). Meanwhile, ROC curves were generated for IKBKE expression. The area under the curve (AUC) of mesenchymal subtype was up to 0.7518 and 0.8127 in the TCGA and CGGA datasets, respectively (Figure 3C,D). To further confirm the IKBKE as a biomarker of the mesenchymal of GBM, a Pearson correlation assay was applied additionally to examine the relationship between IKBKE and subtype biomarkers, as indicated, IKBKE showed a positive correlation with mesenchymal markers (RELB, IL4R, TRADD, CD44, CHI3L1, and TNFRSF1A) and a negative association with proneural markers (SOX2, ASCL1, NKX2.2, OLIG2, DLL3, ERBB3) in both TCGA and CGGA datasets (Figure 3E,F).

### 3.4 Mesenchymal GBM confers an immunosuppressive signature

**FIGURE 4:**
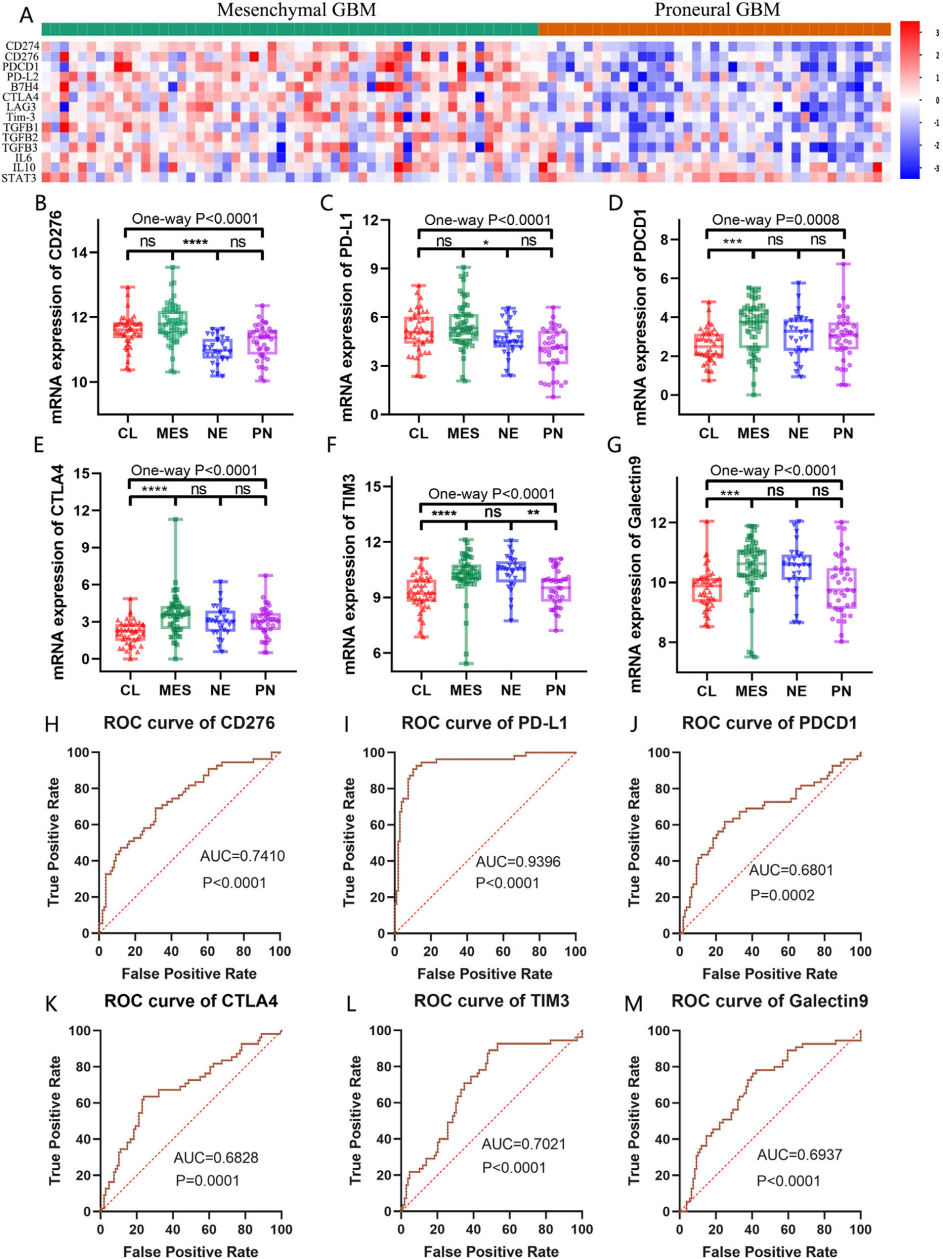
Mesenchymal GBM confers immunosuppressive signature. A, The heatmap compared the gene expression pattern of immunosuppressive factors (immune checkpoints, inflammatory factors) between mesenchymal and proneural subtypes based on TCGA dataset. B‐G, The mRNA expression of immune checkpoints in four subtypes of glioblastoma in TCGA dataset. (ns, not significant, *P < .05, **P < .01, ***P < .001, ****P < .0001). H‐M, ROC curve analyses showed the sensitivity and specificity of immune checkpoints to predict mesenchymal subtype in TCGA dataset

Accumulation of immune checkpoints and inflammatory factors in GBM cells is in response to the resistance of antitumor immunity. To detect the immunosuppressive signature of Mesenchymal GBM, we compared the gene expression pattern of immunosuppressive factors (immune checkpoints, inflammatory factors) between mesenchymal subtype and another typical phenotype, proneural subtype through TCGA‐GBM‐539 mRNA array dataset. The results showed that mesenchymal owns a higher expression of immunosuppressive factors compared to proneural subtype (Figure 4A). Further expression profiles among immune checkpoints in four subtypes of GBM also confirmed this finding. The checkpoints including CD276, PD‐L1, PDCD1, CTLA4, TIM3, and Galectin 9 are all preferentially expressed in mesenchymal subtype (Figure 4B‐G). Meanwhile, those checkpoints are also served as a potential biomarker of mesenchymal subtype with AUC values ranging from 0.6801 to 0.9396 (*P* < .001) (Figure 4H‐M).

### 3.5 IKBKE correlates with inflammation and immune response in mesenchymal GBM

**FIGURE 5:**
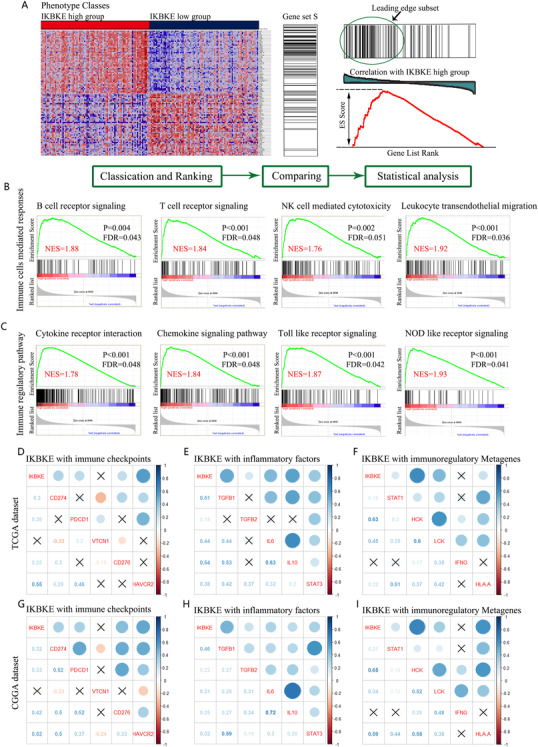
IKBKE correlates with inflammation and immune response in mesenchymal GBM. A, The schematic diagram of Gene Set Enrichment Analysis (GSEA) based on the expression profile of IKBKE in TCGA mesenchymal GBM patients. (Comparing the gene set of IKBKE high group to each of the bins (terms) in the gene ontology). B, GSEA shows the association between IKBKE and immune cells mediated responses in mesenchymal GBM. C, GSEA shows the association between IKBKE and immune regulatory pathways in mesenchymal GBM. Pearson correlation analysis shows the relationship of IKBKE with immune checkpoints, inflammatory factors and immunoregulatory metagenes in mesenchymal GBM based on TCGA dataset (D‐F) and CGGA dataset (G‐I). ×, Not significant

To determine in which biological process IKBKE was mainly implicated in the mesenchymal glioblastoma, GSEA analysis was performed on glioblastoma mRNAseq data from TCGA using C2.p.kegg.v7.0 gene set from the Molecular Signatures Database (MSigDB). The flow diagram of GSEA is showed in Figure 5A. The results indicated that IKBKE in mesenchymal GBM is involved in immune cells mediated responses including B cell and T cell receptor signaling, NK cell mediated cytotoxicity, and leukocyte transendothelial migration, as well as immune regulatory pathways such as cytokine receptor interaction, chemokine signaling pathway, Toll‐like receptor signaling, and Nod‐like receptor signaling (Figure 5B,C). As shown in previous results, mesenchymal GBM is featured with a much more immunosuppressive microenvironment, to further examine the relationship between IKBKE and tumor mediated immunosuppression in gliomas, we analyzed IKBKE in three cohorts, termed, immune checkpoints, inflammatory factors, and immunoregulatory metagenes. Each cohort contained five hub genes, and a Pearson correlation assay was performed between IKBKE and these three cohorts in both TCGA and CGGA datasets. In TCGA GBM dataset (Figure 5D‐F), the results demonstrated that IKBKE is positively associated with immune checkpoints such as CD274, PDCD1, CD276, and HAVCR2 except VTCN1. Moreover, IKBKE is also positively correlated with inflammatory factors (TGFB1, TGFB2, IL6, IL10, and STAT3) and immunoregulatory metagenes (STAT1, HCK, LCK, and HLAA). Similar patterns were also observed in glioma of CGGA dataset (Figure 5G‐I).

### 3.6 IKBKE in mesenchymal GBM is closely associated with JAK‐STAT pathway

**FIGURE 6:**
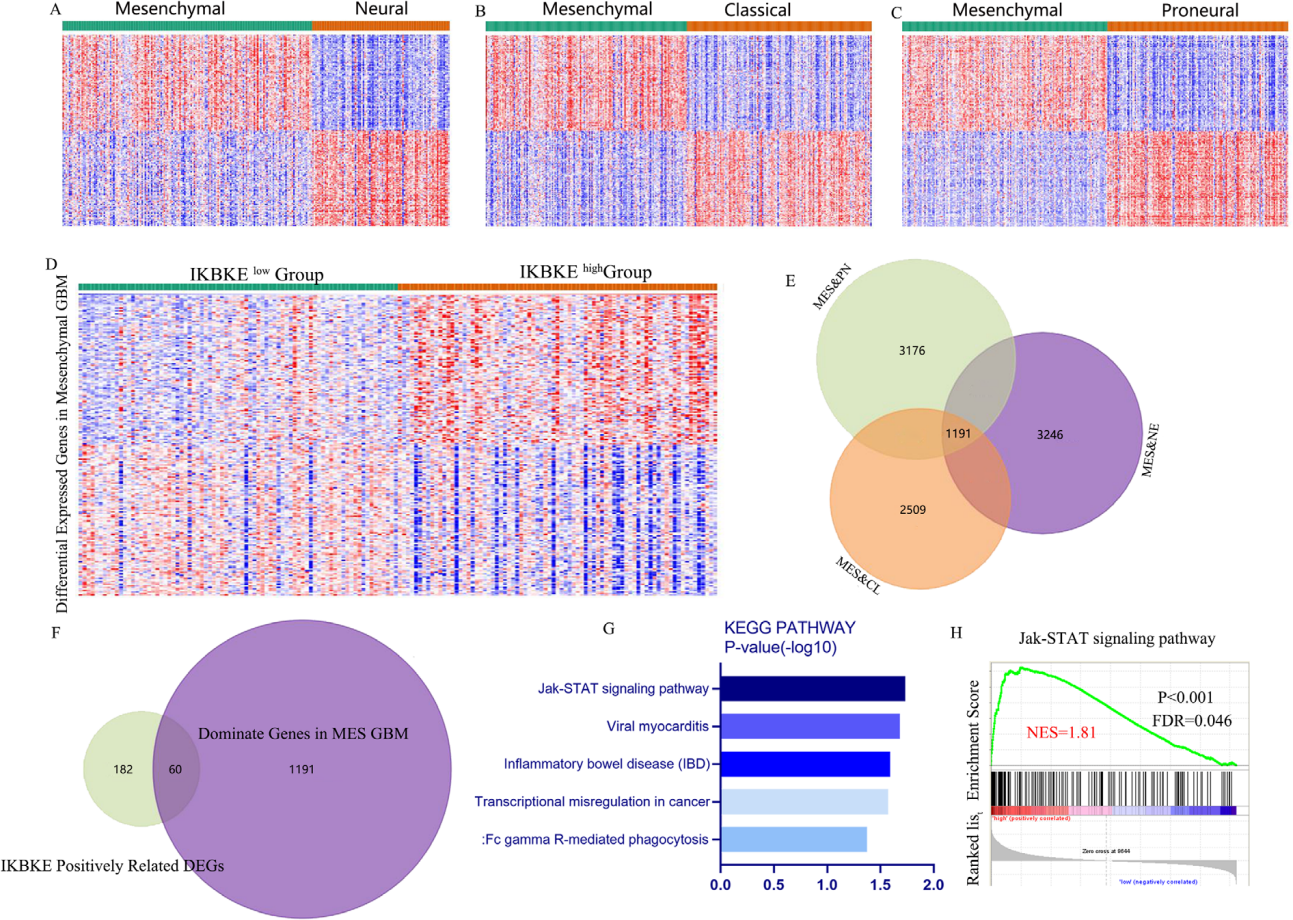
IKBKE is closely associated with to JAK‐STAT pathway in mesenchymal GBM. A, Differentially expressed genes between mesenchymal and Neural GBM. B, Differentially expressed genes between mesenchymal and classical GBM. C, Differentially expressed genes between mesenchymal and proneural GBM. D, Differentially expressed genes between IKBKE high group and IKBKE low group in mesenchymal GBM. E, A comparison of the above DEGs overlapped 1191 common genes specific to the mesenchymal GBM. F, A comparison of IKBKE positively related DEGs in mesenchymal with mesenchymal dominate genes revealed IKBKE associated genes specific to the mesenchymal GBM. G, The most highly correlated processes based on the 60 overlapping genes are plotted by KEGG pathways analysis. H, The enrichment score of JAK/STAT3 pathway in IKBKE high expression mesenchymal GBM patients analyzed by GSEA

To study the underlying gene and pathway pattern that IKBKE regulated in mesenchymal GBM, we employed a series of bioinformatic analyses based on TCGA GBM mRNAarray dataset. First, differentially expressed genes (DEGs) were selected based on Student's *t*‐test to compare the difference between the mesenchymal subtype and the other three GBM subtypes. The threshold of DEGs was determined as follows: *P* ≤ .05, FDR ≤ 0.05, and the top 100 genes were listed in the heatmaps (Figure 6A‐C). Subsequently, we identified 3176 DEGs between mesenchymal subtype and proneural subtype, 3246 DEGs between mesenchymal subtype and neural subtype, and 2509 DEGs between mesenchymal subtype and classical subtype. Next, we compared the above mentioned 3176, 3246, and 2509 genes that overlapped between groups, there yielding a total of 1191 common genes that are specific expressed in mesenchymal GBM (Figure 6E). Finally, we compared the 182 DEGs that positively related to IKBKE^high^ group in mesenchymal GBM (Figure 6D) with 1191 mesenchymal dominate genes, to identify DEGs that were specially involved in the mesenchymal IKBKE^high^ subgroup, which yielded a total of 60 genes (Figure 6F). We uploaded these 60 DEGs for KEGG pathway analysis using the online tool from DAVID software. The outputs were ranked by *P*‐value and the top five items are exhibited (Figure 6G). As shown, the JAK‐STAT is the pathway most specifically associated with IKBKE in mesenchymal GBM. Subsequent Gene Set Enrichment Analysis (GSEA) was applied to validate the tight association between IKBKE and JAK‐STAT signaling in the mesenchymal GBM (Figure 6H).

### 3.7 IKBKE promotes tumor mediated immunosuppression through STAT3/PD‐L1 pathway

**FIGURE 7:**
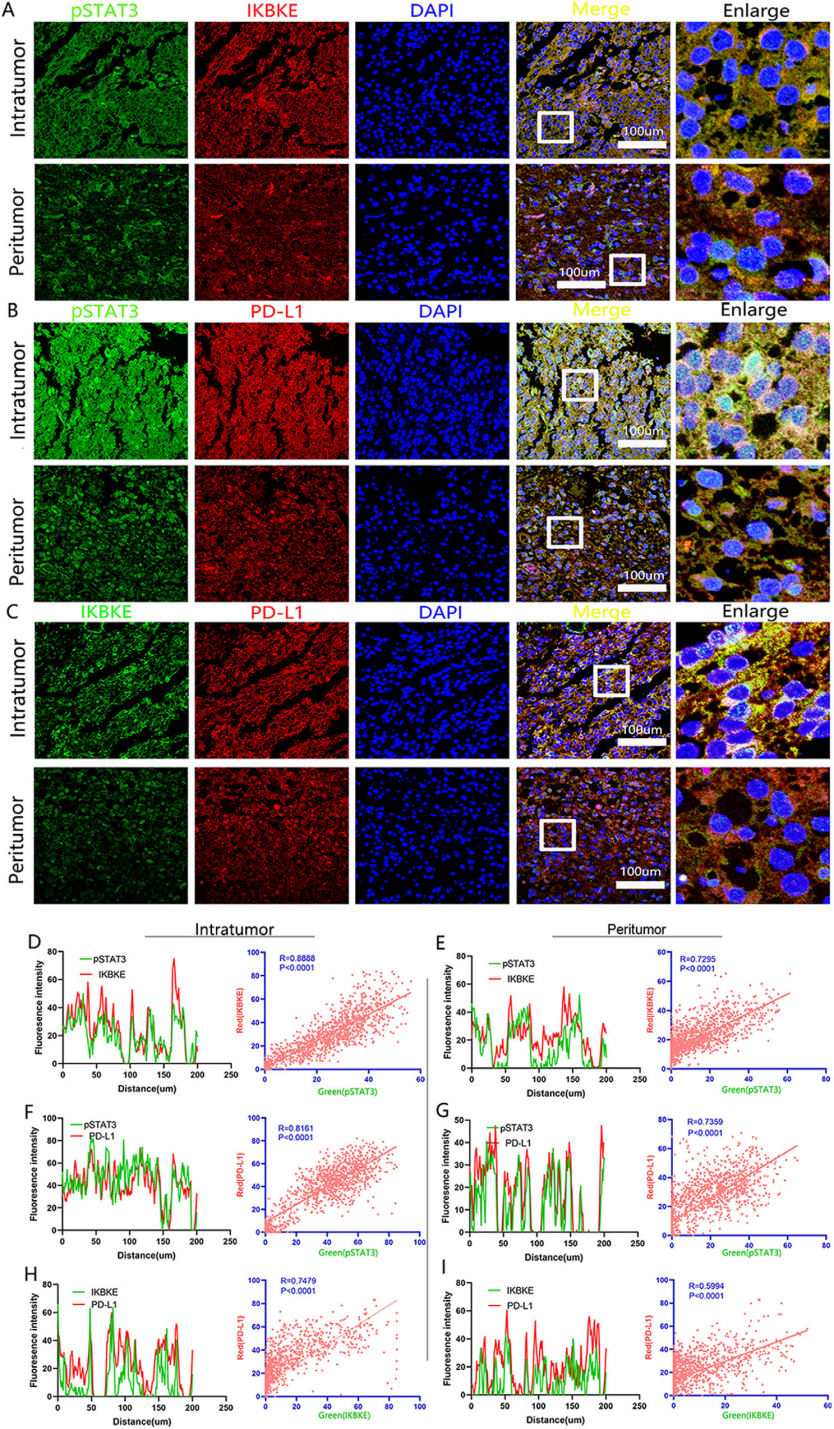
The co‐expression pattern between IKBKE, pSTAT3, and PD‐L1 in GBM tissues. A, Co‐immunofluorescent staining of IKBKE and pSTAT3 in the intratumor and peritumor of GBM tissue. (Scale bar = 100 μm). B, Co‐immunofluorescent staining of pSTAT3 and PD‐L1 in the intratumor and peritumor of GBM tissue. (Scale bar = 100 μm). C, Co‐immunofluorescent staining of IKBKE and PD‐L1 in the intratumor and peritumor of GBM tissue. (Scale bar = 100 μm). D‐I, Colocalization of interested proteins are indicated by the positional overlap curve and analyzed by Pearson correlation analysis

**FIGURE 8:**
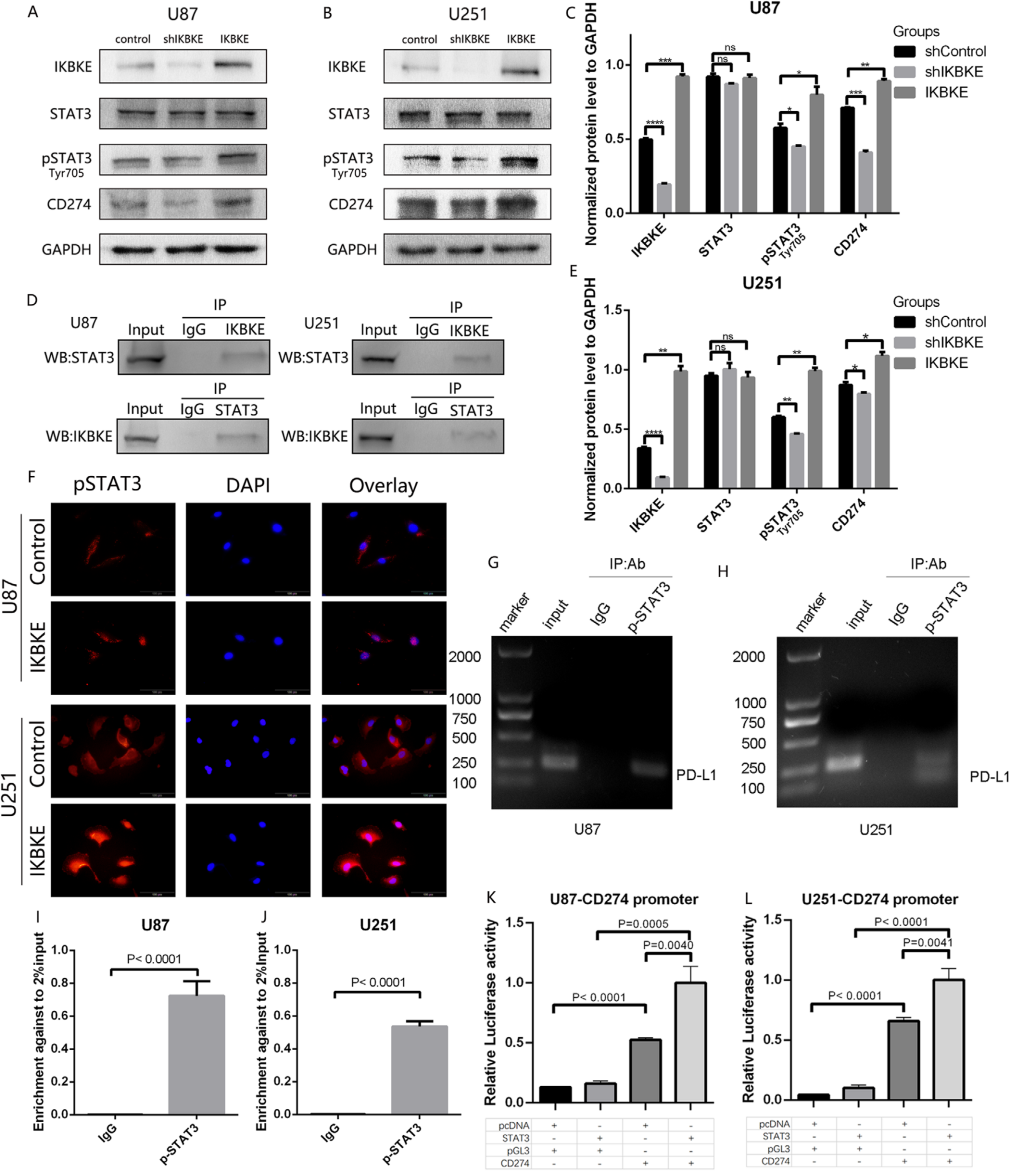
IKBKE promotes tumor mediated immunosuppression through STAT3/PD‐L1 pathway. A,B, Western blotting analysis of IKBKE, STAT3, pSTAT3, and CD274 expression in U87 and U251 glioma cell lines. C,E, The normalized protein expression was shown respectively. (ns, not significant; *P < .05, **P < .01, ***P < .001, ****P < .0001). D, The interaction between IKBKE and STAT3 was validated by co‐IP in U87 and U251 cell lines. F, Overexpression of IKBKE enhances p‐STAT3 nuclear accumulation and STAT3 nuclear translocation validated by immunofluorescence. (Scale bar = 100 μm). G,H, Chromatin Immunoprecipitation‐qPCR results analyzed by agarose gel electrophoresis. (The DNAs were PCR amplified with the primer specific for PD‐L1 promoter and electrophoresed in agarose gel). I,J, Chromatin Immunoprecipitation‐qPCR amplified with the primer specific for PD‐L1 promoter. K,L, Dual‐reporter luciferase assays showed the relative luciferase activity after transfected STAT3‐pcDNA and CD274‐pGL3 plasmids

Based on the results of previous studies, we hypothesized that the JAK‐ STAT pathway may play a critical role in IKBKE mediated immunosuppression of mesenchymal GBM. To determine IKBKE distribution and its correlation with JAK‐STAT signaling and tumor mediated immunosuppression in GBMs, frozen tumor sections were co‐immunostained with pSTAT3 and PD‐L1(CD274) and observed via confocal microscopy (Figure 7A‐C). We found that in cells expressing IKBKE, pSTAT3, and PD‐L1, these proteins were colocalized or adjacent to each other in the same cells within the GBM tissues (Figure 7A‐C). Furthermore, the colocalization curves and Pearson coefficients of intratumor areas displayed a higher co‐expression density of IKBKE, pSTAT3, and PD‐L1 compared to peri‐tumoral GBM tissues (Figure 7D‐I). To further confirm these initial findings, we established IKBKE knockdown and overexpression U87 and U251 glioma cell lines using lentivirus transfection. After silencing IKBKE in U87 and U251, the phosphorylation level of STAT3 and the protein expression of CD274 were decreased according to the western blot assay. Similarly, the overexpression of IKBKE leads to increased protein levels of pSTAT3 and CD274 (Figures 8A‐C and 8E). Furthermore, we performed co‐immunoprecipitation (co‐IP) assays to investigate the interaction of IKBKE with STAT3. The results indicated that IKBKE interacts with STAT3 in both U87 and U251 cell lines (Figure 8D). Meanwhile, we used immunofluorescence staining to investigate whether IKBKE overexpression could increase its nuclear localization. Indeed, the results showed that IKBKE overexpression greatly induces the translocation of STAT3 from cytoplasm to nucleus in two cell lines (Figure 8F). We further applied Ch‐IP and luciferase reporter assay to testify the underlying regulatory mechanism between STAT3 and PD‐L1 (CD274). The results verified the binding relationships of pSTAT3 and CD274 (Figure 8G‐J). Meanwhile, enhancement of STAT3 or CD274 promoter was observed to significantly increase the CD274 transcription level (Figure 8K‐L). Taken together, IKBKE potentiates the phosphorylation and translocation of STAT3 and thus increasing the transcription of CD274.

### 3.8 Silencing of IKBKE suppresses tumor growth in vivo

**FIGURE 9:**
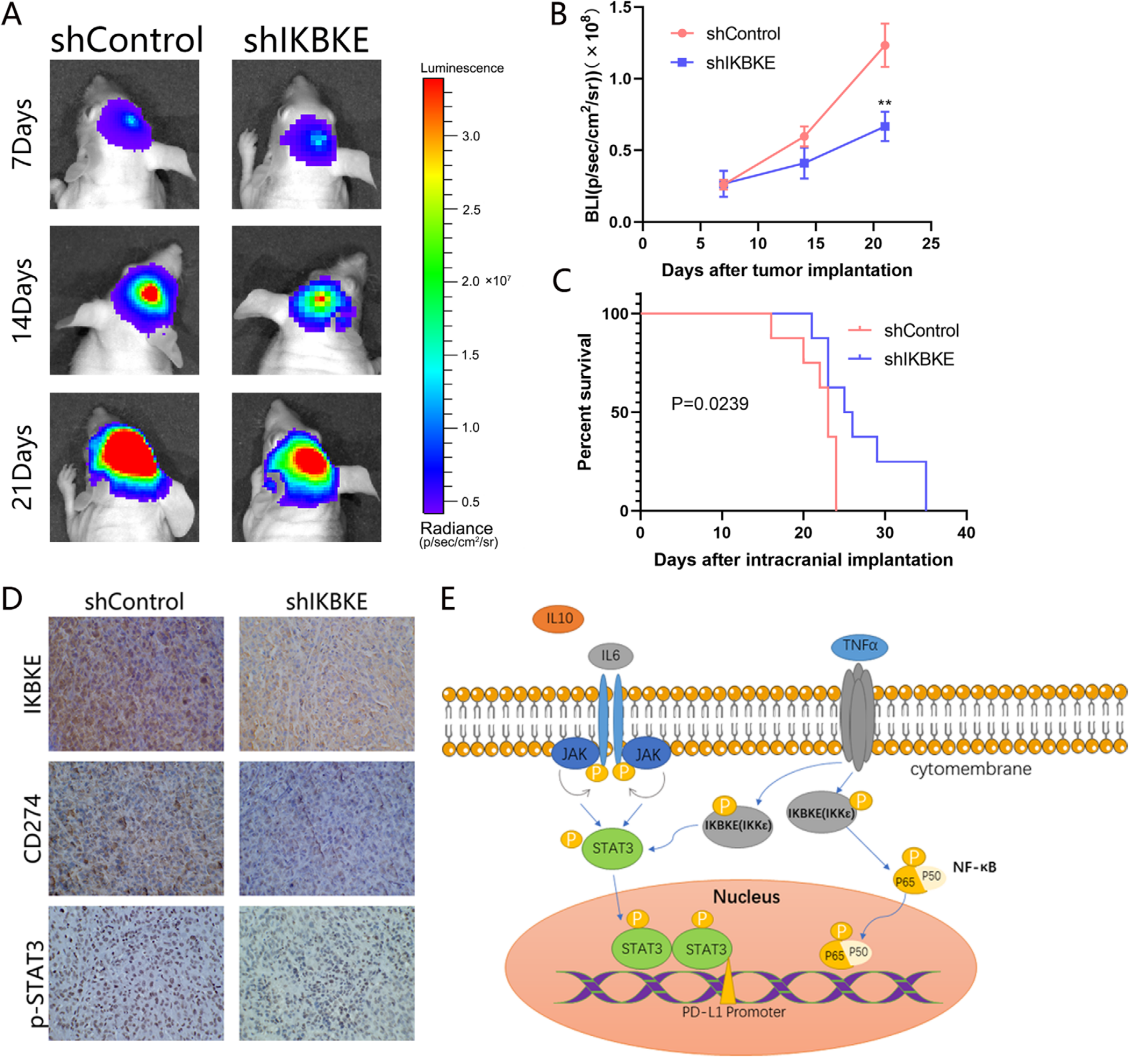
Silencing of IKBKE suppresses tumor growth in vivo. A, The representative bioluminescent images of the tumor‐bearing mice implanted with U87 human glioma cells. B, Quantitative analysis of these bioluminescence images for the shControl and shIKBKE groups. C, The overall survival of mice in the U87‐shControl and U87‐shIKBKE groups. D, Immunohistochemical staining for IKBKE, CD274, and pSTAT3 proteins in IKBKE‐shRNA treated tumors compared to tumors in the shControl group. E, The schematic cartoon of the mechanism of IKBKE as an oncogene positively regulates STAT3/PD‐L1 axis in glioma cells. (**P < .01)

The results from in vivo experiments indicated that silencing of IKBKE inhibits tumor growth. We observed shControl and shIKBKE U87 cells in mouse brains by living imaging system that displayed higher radiance values in line with faster tumoral growth rates (Figure 9A). The results showed that the shIKBKE tumors own lower radiance values than control group on the 7th, 14th, and 21st days after orthotopic xenotransplantation (Figure 9B). Likewise, downregulation of IKBKE linked with a longer survival time (Figure 9C). Moreover, we stained the intracranial tumors with IKBKE, pSTAT3, and CD274. Immunohistochemistry staining showed decreased expression levels of IKBKE, pSTAT3, and CD274 in the shIKBKE group (Figure 9D), which was consistent with the results of in vitro experiments.

## 4 DISCUSSION

Despite the precise molecular classifications have been identified by TCGA working group.^39^, ^40^, ^41^ Three subtypes, namely, proneural (PN), classical (CL), and mesenchymal (MES), appear generally robust phenotypes that are accordant with the classification schemes. Among these subtypes, GBMs in the MES subclass are predominantly primary tumors that develop rapidly de novo and, display a worse outcome than the other two types.^42^, ^43^, ^44^, ^45^, ^46^ A growing number of studies have shed light on the relationship between the subtype classes and the transcriptional network in mesenchymal GBMs.^47^, ^48^, ^49^, ^50^, ^51^ The PN subtype tends to shift toward the MES phenotype upon recurrence or in response to radiotherapy.^31^, ^32^, ^52^, ^53^ This phenomenon is characterized by the increased expression of CD44 and the activation of TNF‐a/NF‐κB signaling.^32^ Hollandf et al demonstrated that CEBPB and STAT3 are the master regulators that responsible for the mesenchymal phenotype transformation in radiation response.^31^ These studies suggest that NF‐κB and STAT3 signaling are the two main regulators that function in the phenotype transformation and maintenance within MES GBM. However, the molecular signatures and mechanisms underlying mesenchymal subtype are still largely unknown. As IKK‐related kinases, both TBK1 and IKBKE are originally known as their ability to regulate NF‐κB signaling and inflammatory responses in addition to canonical IKKs. Among these family members, IKBKE is unique that can coordinately activate both NF‐κB and STAT3 signaling in cancer cells.^9^, ^54^ Thus, it is intriguing that the overexpression of IKBKE in GBM may be the consequence of a functional requirement of mesenchymal phenotype maintenance and immunosuppression in this tumorigenic context.

Critically, our results primarily indicate that IKBKE is highly expressed in glioblastoma and correlates with a poor overall survival. Furthermore, IKBKE is mainly enriched in the necrotic region of the tumor bulk with a mesenchymal subtype preference. IKBKE is associated with tumor recurrence and the immunosuppressive features in mesenchymal GBM. In addition, our studies also uncover a novel mechanism behind IKBKE regulated immunosuppression in GBM, through a series of bioinformatic analyses, we have identified the JAK‐STAT as the most associated signaling pathway that activated in the IKBKE‐mediated mesenchymal GBM. Further cytological studies validate this finding, IKBKE could increase the expression of PD‐L1 through phosphorylation of STAT3. The phosphorylated STAT3 can translocate into nucleus and binding to the PD‐L1 promoter area thus transcriptionally regulating PD‐L1 expression (Figure 9E). The in vivo study further confirmed that knockdown of IKBKE can decrease the tumor growth of GBM and getting a favorable outcome.

## 5 CONCLUSIONS

Our study suggests that IKBKE as the central node in the crosstalk between the NF‐κB and STAT3 signaling within mesenchymal GBM. Targeting GBM through inhibiting IKBKE could restrain not only the proliferation and EMT ability in glioma cells but also the tumor‐mediated immunosuppressive environment. Accordingly, these findings provide basis for the possibility of IKBKE as a promising therapeutic target for glioma treatment.

## AUTHOR CONTRIBUTIONS

Li Yi and Gaochao Guo conceptualized and designed the study. Li Yi, Gaochao Guo, and Jiabo Li performed pathological and biochemical experiments. Li Yi, Xiaoguang Fan, Luqing Tong, Xuya Wang, and Feng Yuan acquired the data and developed the methodology. Li Yi, Jiabo Li, Tao Li, and Peidong Liu performed data analysis and interpretation. Li Yi and Gaochao Guo were associated with writing and revision of the paper. Xuejun Yang, Qiang Huang, and Shengping Yu supervised the study. All authors have reviewed and approved the final manuscript.

## CONFLICT OF INTEREST

The authors have declared no conflict of interest.

## Supporting information


**Supplementary Figure1**. The IHC stain of tissue microarray in glioma patients.

**Supplementary Table1**. Clinical data of glioma patients.

**Supplementary Table2**. The extension of tumor abbreviations in GEPIA.

## Data Availability

The datasets used or analyzed in this study are available from the corresponding author on reasonable request.
