# Supplementary figures and images for: IKBKE, a prognostic factor preferentially expressed in mesenchymal glioblastoma, modulates tumoral immunosuppression through the STAT3/PD‐L1 pathway
Source: Clin Transl Med. 2020 Jul 23;10(3):e130. doi: 10.1002/ctm2.130 (PMC7418810; doi:10.1002/ctm2.130)

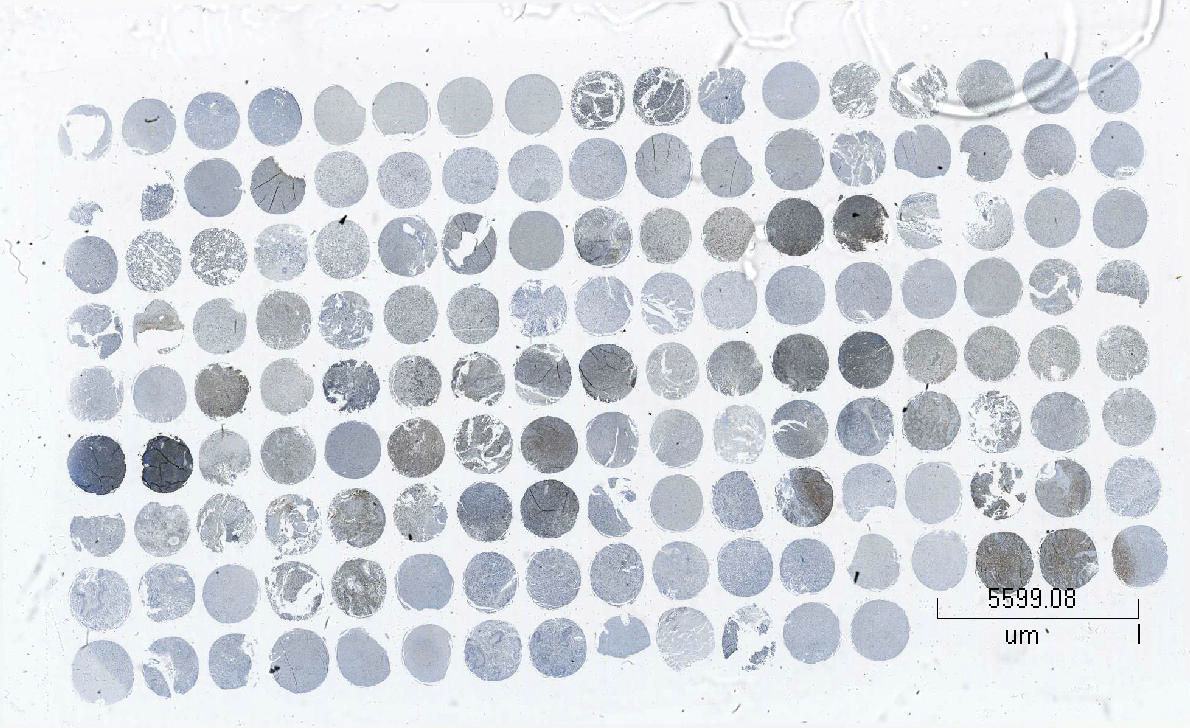

Supplement: Supplementary file 1 — Supplementary Figure1. The IHC stain of tissue microarray in glioma patients. [file CTM2-10-e130-s001.TIF]
